# Impact of pregnancy/childbirth on dispositional optimism in the context of risk of depression, mental health status and satisfaction with life

**DOI:** 10.3389/fpsyt.2023.1271033

**Published:** 2024-01-08

**Authors:** Agnieszka Kułak-Bejda, Andrei Shpakou, Natallia Khvoryk, Liudmila Hutsikava, Ilknur Aydin Avci, Dilek Celik Eren, Lambrini Kourkouta, Areti Tsaloglidou, Konstantinos Koukourikos, Napoleon Waszkiewicz

**Affiliations:** ^1^Department of Psychiatry, Medical University of Białystok, Białystok, Poland; ^2^Department of Integrated Medical Care, Medical University of Białystok, Białystok, Poland; ^3^Department of Obstetrics and Gynecology, Grodno State Medical University, Grodno, Belarus; ^4^Department of Nursing, Faculty of Health Sciences, Ondokuz Mayıs University, Samsun, Türkiye; ^5^Department of Nursing, International Hellenic University, Thessaloniki, Greece

**Keywords:** postpartum, depression, pregnancy, optimism, mental health

## Abstract

**Introduction:**

Life optimism is an inseparable element accompanying every human being. It takes different values depending on the life situation. The present study aimed to measure the level of dispositional optimism in postpartum and pregnant women, compared to women who are not pregnant and have never given birth in Poland, Greece, Turkey, Belarus, and Russia, depending on the level of life satisfaction, risk of depression and mental health.

**Materials and methods:**

A case–control study was carried out among 2017 women, including 584 pregnant women, 528 postpartum women, and 906 women who had never been pregnant and had never given birth (control group) from Poland, Greece, Turkey, Belarus, and Russia.

The study used the LOT-R Life Orientation Test, the Beck Depression Scale (BDI), the Satisfaction With Life Scale (SWLS), the GHQ- 28, and the Edinburgh Postnatal Depression Scale (EPDS) – only in the postpartum group.

**Results:**

Women from the control group showed an average level of optimism, obtaining an average of 16 points in Belarus, 13.4 points in Poland, 13.3 points in Greece, 13.5 points in Turkey, and Russia – 16.3 points. Pregnant women from Belarus had a high level of optimism (17 points), and in other countries, an average level of optimism was in Poland – 14.5 points, Greece – 14.0 points, Turkey – 14.3 points, and Russia – 16.5 points. Women after childbirth had a high level of optimism in Belarus (17.4 points) and Russia (17.2 points), and in other countries had the average level of optimism. In these countries, the lowest level of optimism was found in non-pregnant women. No significant correlation between age and life optimism was found in any group. In Poland, life optimism increased with age in women who had never been pregnant, and in Turkey, in women who were pregnant and after childbirth.

**Conclusion:**

Pregnant women from Belarus had a higher level of optimism than other countries. Non-pregnant women had an average level of optimism. Future studies should include larger groups of women and consider other factors that may additionally contribute to dispositional optimism.

## Introduction

Several terms and gradations of pessimism and optimism may be found in the literature (1–6). Pessimism is a mental attitude in which an undesirable outcome is anticipated from a given situation. Pessimists tend to focus on the negatives of life in general (7).

Optimism is an attitude reflecting a belief or hope that the outcome of some specific endeavor, or outcomes in general, will be positive, favorable, and desirable. For example, the concept of ‘dispositional optimism’, is understood as a way of perceiving the world that involves expecting more positive outcomes (8). They consider it to be a fixed personality trait rather than a variable dependent on the current situation. As such, it can be regarded as an important factor involved in the choice of a goal and in the way it is achieved, determining the effort put into achieving it (8). The terms’ defensive pessimism’ and ‘strategic optimism’ are also distinguished (9, 10).

The development of the above trait is influenced by biological factors (heredity and temperament), and, according to Seligman (11), also by environmental factors (influence of parents, teachers, and early life experiences). Research findings in recent years prove that variables traditionally considered cognitive (self-esteem, satisfaction with life, and optimism) share a common genetic basis (12, 13).

It is worth remembering that life optimism is an intrinsic component that accompanies every human being, although it takes on different magnitudes depending on the life situation and psychological predispositions (5, 11, 12). It determines a person’s behavior, shapes one’s attitude toward oneself, builds high self-esteem and attitudes toward others, determines one’s self- efficacy, and motivates one to take new initiatives. In difficult situations, it reduces tension and stress, facilitates solving difficulties effectively, and triggers adaptive strategies focused on emotions (11). It also facilitates rapid decision-making in a difficult situation for the individual (8). Study results suggest that high levels of dispositional optimism are significantly associated with physical and mental health, life expectancy, high motivation in one’s tasks (even when faced with growing obstacles), positive emotions, more self-efficacy, higher perceived quality of life, resistance to stress, preference for active coping strategies, and a less severe negative impact of stress (8, 14, 15).

The perinatal period, from pregnancy to the first year postpartum, is a transitional period that can result in anxiety and stress for some women. Perinatal anxiety and stress can adversely impact women and children’s physical and psychological health. Understanding these problems is essential to support women better (16).

Numerous studies have been published on the well-known effects of depression during pregnancy and the postpartum period (13, 17, 18). In contrast, the relationship between obstetrical outcomes and dispositional optimism is still debated.

Life satisfaction can be reduced by lower optimism. For example, it is suggested that increasing optimism could reduce the negative consequences of activities of daily living limitations on life satisfaction among middle-aged older adults (19).

Patients’ optimism is correlated with a lower risk of depression and anxiety. A study by Rodrigues et al. (20) demonstrated that high anxiety and depression are correlated with poor optimism and quality-of-life scores.

Antenatal depression is a debilitating experience for many women with significant personal and familial sequelae. Optimism is inversely correlated with depression and directly correlated with improved birth outcomes (21).

Optimism is a potentially modifiable variable that could be used to design antenatal prevention and treatment programs. There are few studies on dispositional optimism in postpartum and pregnant women in Poland (22). Moreover, no similar studies were conducted in Belarus, Greece, Turkey, or Russia. Therefore, we wanted to compare the impact of pregnancy and the postpartum period on dispositional optimism in relation to life satisfaction, risk of depression, and mental health between these different countries with different cultures.

### Aim of the study

The study was designed to measure the level of dispositional optimism in postpartum and pregnant women, compared to women who were not pregnant at the time of the survey and had never given birth in Poland, Greece, Turkey, Belarus, or Russia in relation to satisfaction with life, risk of depression and mental health status.

The research hypotheses were that (1) postpartum and pregnant women, compared to women who are not pregnant and have never given birth (control group), show higher levels of optimism; (2) in all study groups/countries, women who had higher optimism levels also had higher satisfaction with life, lower risk of depression, and better mental health.

## Materials and methods

A case–control study was carried out among 2017 women surveyed, including 584 pregnant women, 528 postpartum women, and 906 nulliparous women (the control group) from Poland, Greece, Turkey, Belarus, and Russia. The sample selection was purposive. A purposive sample is a non-probability sample selected based on population characteristics and the study’s objective. Purposive sampling differs from convenience sampling and is also known as judgmental, selective, or subjective sampling. The pregnant women were recruited from Obstetrics Departments, the postpartum women were from GP clinics, and the control group included women students and workers of the Universities from the studied countries. Written informed consent for participation in the study was obtained from all participants. The study was retrospective, and participation was voluntary and anonymous. Women were included in the study if they were over 18 years of age, willing to give informed consent, and could communicate in the mother language. The questionnaires were given to the participants by the authors of the study. The authors from Belarus, Russia, Greece, and Turkey have sent the filled questionnaires in their native languages to Poland. Next, the data was then entered into a spreadsheet by a statistician. All the question-and-answer numbers were the same in all five countries. This made the statistician able to analyze the results easily.

The Bioethics Committee APK.002.587.2021 approved the Medical University of Białystok, Poland study. Surveys were collected between November 2021 and December 2022. The study used the following questionnaires:

Beck Depression Inventory (BDI) to assess the presence and severity of depressive symptoms (23) – all groups. It is used to self-assess the presence and severity of depression symptoms. The scale consists of 21 points rated according to the intensity of symptoms, from 0 to 3. For each point, the respondent should select one answer that, in his opinion, best describes his condition in the indicated period (before the doctor asks the patient to complete the scale, he should specify what period the answers should refer to – a month, a week or the last 24 h)—obtaining from 0 to 11 points indicates no depression, 12–26 points with a mild depressive episode, 27–49 points - with a moderate depressive episode and 50–63 points – a major depressive episode. The discriminatory power coefficients (corrected item-scale correlation coefficients) of the items were: in the control group from 0.48 to 0.70 (*M* = 0.59, Me = 0.61) and in the clinical group from 0.38 to 0.79 (*M* = 0.66, Me = 0.69). The Cronbach’s alpha coefficient was 0.93 and 0.95 for the entire scale, respectively.

Edinburgh Postnatal Depression Scale – EPDS (24) – a group of postpartum women.

The Edinburgh Postnatal Depression Scale was developed to assist health professionals in detecting mothers suffering from postpartum depression, a distressing disorder more prolonged than the “blues” (which can occur in the first week after delivery). The scale consists of 10 questions, each with—four ready-made answers. The woman is asked to read each statement and choose the one that best reflects how she has felt over the past 7 days. The maximum number of points is 30. Mothers scoring above 12 or 13 are likely to be suffering from depression and should seek medical attention, sensitivity, and specificity at the level of 84.2–93.9% and 75.2–76.7%, and Cronbach’s alpha – 0.87–0.88%.

Satisfaction With Life Scale – SWLS (25, 26) – all groups. The scale contains five statements. The respondent assessed the extent to which each of them related to his/her life so far, where: 1 – meant – I completely disagree, 2 – I do not agree, 3 – I rather disagree, 4 – I neither agree nor disagree, 5 – I rather agree, 6 – I agree and 7 – I completely agree. The obtained ratings were added up, and the overall result indicated satisfaction with one’s own life. The range of results could be from 5 to 35 points, and the higher the result, the greater the sense of satisfaction with life: 5–9 points. – a person dissatisfied with their life, 10–14 points, a person very dissatisfied with their life, 15–19 points. – person rather dissatisfied with their life, 20 points. – person neither satisfied nor dissatisfied with their life, 21–25 points. – person rather satisfied with their life, 26–30 points. – a person very satisfied with their life, 31–35 points. – a person satisfied with their life. Results between 1 and 4 sten were treated as low results, and between 7 and 10 sten as high results. Results within 5 and 6 sten are considered average. The reliability index (Cronbach’s alpha) of the SWLS, established in a study of 371 adults, is 0.81. The scale constancy index, determined by testing a group of 30 people twice with an interval of 6 weeks, was 0.86.

General Health Questionnaire GHQ-28 (27) – all groups. The scale is used to assess the state of mental health and allows for the identification of people whose mental state has undergone a temporary or long-term breakdown as a result of experienced difficulties, problems, or as a result of mental illness and those who are at significant risk of mental health disorders. The questionnaire consists of 28 questions measuring four symptom areas: somatic disorders (A), anxiety (B), functional disorders (C), and depression (D). Theoretical scores for the scales range from 7 to 28 points; the higher the score, the greater the difficulties experienced. Cronbach’s alpha coefficients are 0.955, 0.956, 0.945, and 0.926, respectively. The GHQ-28 scale examines four dimensions of mental state: A – somatic symptoms (Cronbach’s alpha for the study group = 0.876), B – anxiety and insomnia (alpha = 0.916), C – social functioning disorders (alpha = 0.933), D – symptoms of depression (alpha = 0.941), which together give an overall score.

Life Orientation Test-Revised LOT-R (25) – all groups. Life Orientation Test-Revised (LOT- R), developed by Scheier, Carter, and Bridges in the Polish adaptation of Poprawa and Juczynski (25), is used to measure dispositional optimism expressing generalized expectations concerning positive events. It is intended for healthy and unhealthy people. It consists of 10 statements, six of which have a diagnostic value for dispositional optimism. The overall test result is the sum of the evaluation of six statements, including three positive and three negative. Possible results are in the range of 0–24 points. The higher the score, the higher the level of optimism. The reliability determined by Cronbach’s alpha coe_cient is 0.78 for the original version and 0.76 for the Polish version.

### Statistical analysis

The Statistica 13.0 PL program was used for statistical calculations. The Spearman’s rank correlation coefficient was used for the analysis, and the analysis was done by country and group concerning being pregnant or postpartum. Due to the large number of correlations analyzed (the analysis was done by country and study group), the exact *p*-values of the test probabilities were omitted, only denoting the level of statistical significance of a given correlation with the symbol *. The tables use a color scheme to facilitate interpretation of the results. Due to the normal distribution of the LOT-R measure, the t-test for independent samples was used in the analysis. Statistical significance was evaluated at *p* < 0.05.

## Results

Table 1 illustrates a summary of the demographics of the respondents by status: not pregnant, pregnant, or postpartum. Among the surveyed women, the group of pregnant (8.1%) and postpartum women from Russia were very low (9.1%) and the highest from Poland (29.6 and 30.9% respectively). Significant differences were found in the residence structure of surveyed women from different countries, which probably depends on the geographical region where the surveys were conducted. Therefore, it seems that it can be assumed that in the 21st century, the place of residence does not significantly determine the quality of life, and the influence of this factor should not be considered further.

**Table 1:** Demographics of the respondents by status: not pregnant, pregnant, postpartum.

Group	Country – group size				
	Belarus	Poland	Greece	Turkey	Russia
Control *N* = 906	136 (15.0%)	131 (14.5%)	144 (15.9%)	150 (16.6%)	345 (38.0%)
Pregnant *N* = 584	147 (25.2%)	173 (29.6%)	114 (19.5%)	103 (17.6%)	47 (8.1%)
Postpartum *N* = 527	103 (19.5%)	163 (30.9%)	106 (20.1%)	107 (20.3%)	48 (9.1%)
Total *N* = 2017	386 (19.1%)	467 (23.2%)	364 (18.0%)	360 (17.8%)	440 (21.9%)

Group	Country	Age [years]						
		$\bar{x}$	Me	*s*	*c*_25_	*c*_75_	Min.	Max.
Control	Belarus	19.2	19	2.0	18	20	17	30
	Poland	33.5	33	9.0	26	39	18	50
	Greece	21.0	20	4.0	19	21	18	50
	Turkey	28.3	26	8.1	22	33	17	49
	Russia	18.5	18	1.1	18	19	17	24
Pregnant	Belarus	29.2	29	5.5	25	33	18	40
	Poland	30.4	30	5.0	27	34	20	43
	Greece	34.7	33	8.9	27	43	19	50
	Turkey	27.7	27	5.3	24	31	17	41
	Russia	27.7	29	4.6	24	30	17	37
Postpartum	Belarus	28.7	29	5.3	25	32	18	42
	Poland	31.0	30	5.4	27	35	20	45
	Greece	37.6	36	8.8	30	45	19	50
	Turkey	28.1	28	4.7	25	31	19	44
	Russia	28.1	28	4.3	24.5	31.5	18	37

	Place of residence					Total *N* = 2017
	Belarus *N* = 386	Poland *N* = 467	Greece *N* = 364	Turkey *N* = 360	Russia *N* = 440	
Rural	5.8%	33.5%	28.1%	16.7%	7.6%	18.0%
City with a population < 5,000	2.9%	6.1%	12.8%	0.0%	5.3%	5.3%
City with a population of 5,000–25,000	10.6%	12.9%	11.9%	0.3%	10.1%	9.2%
City with a population of 25,000–40,000	4.0%	10.7%	8.0%	19.8%	2.7%	8.7%
City with a population of 40,000–100,000	4.5%	13.6%	12.5%	26.2%	6.9%	12.2%
City with a population of 100,000–200,000	4.2%	3.5%	4.8%	8.9%	5.9%	5.3%
City with a population > 200,000	68.0%	19.7%	21.9%	28.1%	61.6%	39.4%
No answer	2.0%	2.1%	3.3%	0.3%	0.7%	1.7%

In Poland, respondents with a master’s degree dominated (201 people), and in Turkey with a bachelor’s degree (155 people). Most respondents from Belarus (114 people), Greece (116 people), and Russia (262 people) were students. The results are shown in Table 2.

**Table 2:** Educational background of respondents.

Education	Belarus *N* = 386			Poland *N* = 467			Greece *N* = 364			Turkey *N* = 360			Russia *N* = 440			Total
	1	2	3	1	2	3	1	2	3	1	2	3	1	2	3	
	*N* = 136	*N* = 147	*N* = 103	*N* = 131	*N* = 173	*N* = 163	*N* = 144	*N* = 114	*N* = 106	*N* = 150	*N* = 103	*N* = 107	*N* = 345	*N* = 47	*N* = 48	
Vocational	4	41	37	7	11	11	1	18	15	51	36	42	5	16	22	317
Total	82			29			34			129			43			
Bachelor’s degree	2	35	16	33	28	23	27	33	32	74	40	41	4	12	8	408
Total	53			84			92			155			24			
Master’s degree	9	40	23	42	75	84	3	5	8	18	8	11	9	6	13	354
Total	72			201			16			37			28			
Student	92	12	10	14	10	13	109	3	4	1	1	13	250	10	2	544
Total	114			37			116			15			262			
Secondary education	29	19	17	35	49	32	4	55	47	6	18	0	77	3	3	394
Total	65			116			106			24			83			

1, Control group; 2, pregnant women; 3, postpartum women.

The study used the Life Orientation Test (LOT-R) to assess how women’s optimism varied by status pregnant, postpartum, not pregnant, and the control group across different countries. Pregnant, postpartum, and non-pregnant women had average or medium optimism. We observed the differences in the scoring optimism between the studied countries. Women from Belarus and Russia were more optimistic than other countries.

Women in the control group showed average optimism, scoring 16 points in Belarus, 13.4 points in Poland, 13.3 points in Greece, 13.5 points in Turkey, and 16.3 points in Russia. Pregnant women from Belarus showed high optimism, at 17 points on average, and other countries showed medium optimism, scoring 14.5 points in Poland, 14.0 points in Greece, 14.3 points in Turkey, and 16.5 points in Russia. In contrast, postpartum women showed high optimism in Belarus, at 17.4 points on average, and Russia, at 17.2 points on average. The average optimism in the other countries was Poland at 14.1 points, Greece at 14.5 points, and Turkey at 14.0 points.

**Figure 1:**
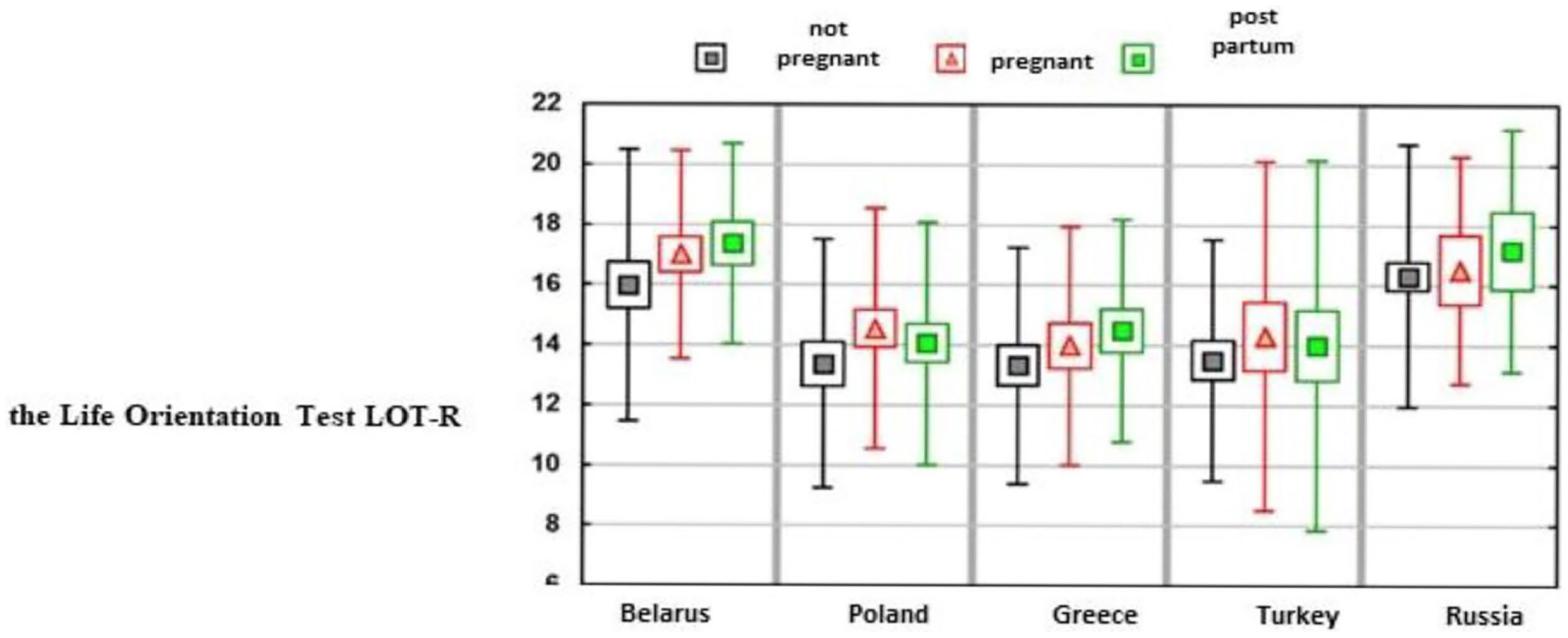
Assessment of the study groups using the *Life Orientation Test LOT-R*.

It only occurred in Russia that being pregnant or giving birth did not affect optimism. In the remaining countries, differences were statistically significant (in Belarus, marginally above the significant score limit: *p* = 0.0594). Analysis of the mean or median values revealed that in each of these countries, the lowest optimism was characterized by women who were not pregnant (Table 3 and Figure 1).

**Table 3:** Assessment of the study groups using the *Life Orientation Test LOT-R*.

Group	LOT-R														
	Belarus			Poland			Greece			Turkey			Russia		
	$\bar{x}$	Me	IQR	$\bar{x}$	Me	IQR	$\bar{x}$	Me	IQR	$\bar{x}$	Me	IQR	$\bar{x}$	Me	IQR
Not pregnant	16.0	16	6.0	13.4	13	5.0	13.3	13	5.0	13.5	14	4.0	16.3	17	5.0
Pregnant	17.0	17	4.0	14.5	14	5.0	14.0	14	5.0	14.3	16	8.0	16.5	17	3.5
Postpartum	17.4	18	4.0	14.1	14	6.0	14.5	15	4.0	14.0	16	11.0	17.2	18	5.5
*p*	0.0594			0.0353*			0.0655			0.0465*			0.5724		

Correlations between optimism by the LOT-R scale and depressive symptoms, life satisfaction, and mental health were examined (Table 4). Generally, we found positive correlations between optimism and life satisfaction and negative correlations between optimism and depressive symptoms and mental health in the studied women. In detail, in the non-pregnant women’s group – LOT-R was positively correlated with life satisfaction and negatively correlated with depressive symptoms and mental health. However, most correlations were at a weak or average level. Among pregnant women, similar correlations were only found in Turkey. The correlations in the other countries are much weaker, and in Russia and Belarus, the LOT-R measures only correlated with life satisfaction. The correlations analyzed in postpartum women were similar, except they were significantly stronger in Turkey.

**Table 4:** Correlations of LOT-R measures with BDI, EPDS, SWLS and GHQ-28 measures.

Measures of depression, mental distress and quality of life	Life orientation test (LOT-R) and other psychometric measures				
	Belarus	Poland	Greece	Turkey	Russia
Not pregnant women					
BDI	−0.57***	−0.35***	−0.48***	−0.41***	−0.48***
SWLS	0.58***	0.29***	0.39***	0.31***	0.43***
GHQ (somatic symptoms)	−0.39***	−0.25**	−0.06	−0.29***	−0.29***
GHQ (anxiety, insomnia)	−0.39***	−0.30***	−0.30***	−0.36***	−0.40***
GHQ (dysfunctions)	−0.35***	−0.41***	−0.29***	−0.34***	−0.39***
GHQ (depressive symptoms)	−0.45***	−0.34***	−0.39***	−0.34***	−0.36***
GHQ (total score)	−0.44***	−0.36***	−0.31***	−0.41***	−0.42***
Pregnant women					
BDI	−0.03	−0.13	−0.31**	−0.48***	−0.38*
SWLS	0.34***	0.35***	0.39***	0.52***	0.20
GHQ (somatic symptoms)	0.11	−0.23**	−0.16	−0.39***	0.01
GHQ (anxiety, insomnia)	0.01	−0.26***	−0.16	−0.43***	−0.07
GHQ (dysfunctions)	−0.04	−0.16*	−0.13	−0.36***	0.16
GHQ (depressive symptoms)	0.01	−0.21**	−0.27**	−0.44***	−0.28
GHQ (total score)	0.04	−0.29***	−0.16	−0.53***	−0.01
Postpartum women					
BDI	−0.31**	−0.22**	−0.45***	−0.63***	−0.34*
EPDS	−0.25*	−0.44***	−0.37***	−0.57***	−0.10
SWLS	0.23*	0.33***	0.43***	0.66***	0.08
GHQ (somatic symptoms)	−0.07	−0.08	−0.27**	−0.69***	0.09
GHQ (anxiety, insomnia)	−0.06	−0.14	−0.32**	−0.50***	−0.01
GHQ (dysfunctions)	−0.12	−0.19*	−0.19	−0.51***	0.02
GHQ (depressive symptoms)	−0.09	−0.32***	−0.25*	−0.62***	−0.01
GHQ (total score)	−0.11	−0.17*	−0.32***	−0.66***	0.03

A partial confirmation of the hypotheses was obtained. A series of graphs shows the correlations between BDI and LOT-R for each country and each woman’s status. It examined how the level of life optimism is related to selected factors. First, the influence of age on the level of life optimism was analyzed – the analysis was carried out taking into account the specificity of the country and belonging to a group.

The results turned out to be quite varied, the most important being that:

- In none of the groups of women from Belarus, Greece, and Russia, a statistically significant relationship between age and the level of life optimism found;
- Among the Polish women, the level of life optimism increased with age, only in women who had never been pregnant;
- Age was also a factor causing an increase in optimism in life in Turkey, and the correlations concerned pregnant women, especially after childbirth (*r* = 0.50, so the correlation here is at an average level). The results are presented in Table 5.

**Table 5:** Correlations of LOT-R measures with age and marital status of women.

Country	Group		
	Not pregnant	Pregnant	Postpartum
	Correlation of LOT-R and women’s age		
Belarus	−0.07 (*p* = 0.4246)	0.10 (*p* = 0.2612)	−0.19 (*p* = 0.0843)
Poland	0.32 (*p* = 0.0003***)	0.08 (*p* = 0.2924)	0.11 (*p* = 0.1822)
Greece	−0.06 (*p* = 0.5030)	0.18 (*p* = 0.0530)	0.17 (*p* = 0.0914)
Turkey	0.08 (*p* = 0.3028)	0.29 (*p* = 0.0029**)	0.50 (*p* = 0.0000***)
Russia	−0.06 (*p* = 0.2955)	0.13 (*p* = 0.3961)	−0.09 (*p* = 0.5912)

Correlation of LOT-R (mean with 95% confidence interval) and marital status					
Not pregnant					
Lonely	16.3 (15.5; 17.1)	13.1 (12.0; 14.2)	13.3 (12.5; 14.1)	13.0 (12.1; 13.8)	16.3 (15.8; 16.8)
In relation with	12.9 (10.3; 15.6)	13.7 (12.7; 14.7)	13.5 (12.1; 14.9)	14.4 (13.3; 15.4)	16.1 (14.0; 18.2)
*p*	0.0085**	0.4551	0.7913	0.0383*	0.8397
Pregnant					
Lonely	15.9 (13.7; 18.1)	14.1 (12.8; 15.4)	14.5 (11.4; 17.6)	×	14.0 (10.7; 17.3)
In relation with	17.1 (16.5; 17.7)	14.7 (14.0; 15.4)	13.9 (13.2; 14.7)	14.4 (13.2; 15.5)	16.8 (15.6; 18.1)
*p*	0.2968	0.4452	0.6242	×	0.1147
Postpartum					
Lonely	16.3 (14.0; 18.6)	13.3 (11.7; 14.8)	14.2 (11.7; 16.6)	×	18.0 (×)
In relation with	17.5 (16.7; 18.3)	14.2 (13.5; 15.0)	14.4 (13.6; 15.2)	14.0 (12.8; 15.2)	17.2 (15.8; 18.5)
*p*	0.2608	0.3041	0.8608	×	×

×−all or almost all of the women were in a relationship, which made some calculations impossible.

Married and unmarried women dominated the surveyed population, and the remaining groups were much smaller; therefore, to ensure the appropriate size of the compared groups, the groups of single and married women were combined, and the analysis was conducted in such a system. Due to the normal distribution of the LOT-R measure, the t-test for independent samples was used in the analysis.

In the group of pregnant and postpartum women, in those countries where a comparison was possible (because, for example, in Turkey, and partly also in Russia, all or almost all women were in a relationship), no significant differences in optimism were found. On the other hand, in the group of non-pregnant women from Belarus, single people had a significantly higher level of optimism. A statistically significant difference was also found among Turkish women who were not pregnant but in favor of women in a relationship who had a slightly higher level of life optimism.

Another analysis concerned the level of life optimism depending on having children. In none of the countries, having children by pregnant women did not affect their current level of life optimism (Table 6).

**Table 6:** Correlations of LOT-R measures with the fact of having children.

Posiadanie dzieci	LOT-R (mean with 95% confidence interval) PREGNANT WOMEN				
	Belarus	Poland	Greece	Turkey	Russia
tak	17.2 (16.4; 18.0)	14.6 (13.7; 15.4)	14.1 (13.3; 15.0)	14.6 (12.7; 16.4)	15.9 (13.6; 18.2)
nie	16.8 (16.0; 17.7)	14.5 (13.6; 15.5)	13.2 (11.4; 15.1)	14.1 (12.7; 15.6)	17.0 (15.7; 18.2)
*p*	0.5205	0.9603	0.3955	0.7019	0.3618
Country		Life Orientation Test (LOT-R) a number of children PREGNANT WOMEN			
Belarus		0.05 (*p* = 0.5614)			
Poland		−0.02 (*p* = 0.8141)			
Greece		0.01 (*p* = 0.9516)			
Turkey		0.03 (*p* = 0.7450)			
Russia		−0.07 (*p* = 0.6657)			

The analysis was also performed considering the number of children already had, using Spearman’s rank correlation analysis. However, the correlation analysis also showed no relationship between pregnant women’s level of life optimism and the number of children they had.

The type of childbirth did not influence the level of life optimism significantly. The level of optimism of pregnant women with a natural or artificial miscarriage did not differ significantly from those of other women. The only difference concerned pregnant women from Turkey – those with a natural miscarriage had a significantly lower level of life optimism (11.9 vs. 15.9 points; *p* = 0.0392*). The results are shown in Table 7.

**Table 7:** Correlations of LOT-R measures in the group of pregnant women with the type of delivery and miscarriage.

	LOT-R (mean with 95% confidence interval) PREGNANT WOMEN				
	Belarus	Poland	Greece	Turkey	Russia
	Type of childbirth				
Natural childbirth	16.9 (15.9; 17.9)	14.4 (13.1; 15.7)	14.3 (13.2; 15.3)	15.4 (12.5; 18.3)	15.8 (13.1; 18.6)
Cesarean section	18.0 (16.5; 19.6)	14.2 (12.9; 15.4)	13.4 (12.2; 14.7)	14.0 (11.5; 16.6)	15.8 (6.3; 25.2)
*p*	0.2021	0.8097	0.3402	0.4747	0.9728
Going through a natural miscarriage					
Yes	17.6 (16.1; 19.1)	15.0 (13.4; 16.6)	14.3 (12.5; 16.0)	11.9 (7.4; 16.3)	15.5 (8.8; 22.2)
No	17.4 (16.5; 18.3)	14.1 (13.0; 15.2)	13.9 (13.0; 14.9)	15.9 (14.1; 17.7)	15.9 (12.9; 18.9)
*p*	0.8409	0.4033	0.7073	0.0392*	0.8806
Going through an artificial miscarriage					
Yes	17.9 (15.7; 20.2)	14.5 (0.0; 46.3)	15.9 (13.2; 18.6)	14.0 (9.8; 18.2)	15.3 (0.0; 34.6)
No	17.3 (16.4; 18.1)	14.2 (13.3; 15.2)	13.9 (13.0; 14.7)	14.8 (12.6; 17.0)	15.9 (13.5; 18.4)
*p*	0.4986	0.9317	0.1421	0.7339	0.8494

## Discussion

Our study contributes new knowledge about dispositional optimism in pregnant and non-pregnant women from five different cultural countries: Poland, Belarus, Greece, Russia, and Turkey.

Women in the control group showed average optimism. Similar results have been obtained in pregnant and postpartum women except for women from Belarus who had high optimism. The dispositional optimism in the LOT-R scale was positively correlated with life satisfaction in the studied and the control group. In contrast, dispositional optimism was negatively correlated with depressive, somatic, and anxiety symptoms. The dispositional optimism was positively correlated with age among Polish and Turkish women. Having children does not affect the level of optimism.

Our findings are in agreement with previous results (21, 22, 28).

Niewiadomska et al. (22) determined the role of dispositional optimism in the relationship between health locus of control and self-efficacy in pregnant women with threatened preterm labor. For women with a high-risk pregnancy, dispositional optimism was a significant resource for coping with their problems. Optimistic pregnant women maintained a positive outlook, even when confronted with difficult, negative experiences such as threatened preterm labor.

In a study from New Zealand, women in hospital and community groups completed a battery of questionnaires on pregnancy and health history, life events, anxiety, optimism, coping, and relationship factors (28). The groups did not differ on life events, optimism, and coping self-efficacy. Predictors of acute anxiety differed across the groups: for hospitalized women, anxiety was predicted by the rating of their health and their dispositional optimism; for women in the community, anxiety was predicted by stressful life events, dispositional optimism, and coping self-efficacy.

In a prospective cohort study (29) completed between 5/2019-2/2022, nulliparous pregnant women completed a validated assessment of dispositional optimism at <20 weeks’ gestation and were followed until delivery. The authors demonstrated that lower early- pregnancy ispositional optimism was associated with significantly higher odds of adverse maternal outcomes.

Italian, Canadian, German, and Japanese studies confirm that positive orientation can be considered a ‘good functioning syndrome’ that correlates positively with the health status assessment (30). Dispositional optimism has been associated with protection against depressive symptoms and reduced negative affect in multiple contexts (31).

The transition to motherhood is a time of elevated risk for clinical depression. Dispositional optimism may be protective against depressive symptoms; however, the arrival of a newborn causes numerous challenges that may contribute to depressed mood. Robakis et al. (32) explored the relative contributions of antenatal and postnatal optimism regarding maternity to depressive symptoms in the postnatal period. Pregnant women underwent clinician interviews in the third trimester to record psychiatric history and antenatal depressive symptoms and administer a novel measure of optimism toward maternity. The authors concluded that antenatal optimism is the approach that is most protective against postnatal depressive symptoms and that this is true irrespective of either mood disorder history or parity.

Kazmierczak et al. (33) analyzed risk factors for perinatal mental disorders and assessed dispositional optimism in women at 37 weeks pregnant and 6 weeks after giving birth. The authors demonstrated that the main predictors of perinatal mental disorders were the variables: mental health disorders before pregnancy, subjectively reported decreased mood in pregnancy, the lack of social support, and the tendency to pessimism.

The literature emphasizes that optimism affects human health in multiple ways (34, 35). Firstly, it improves the functioning of the immune system (when one has a negative disposition toward the world, negative emotions tend to dominate one’s life, resulting in lower levels of catecholamines and endorphins, which translates into a weaker immune system and greater susceptibility to infection). Secondly, people who are positive believe that they have control over their lives and are more willing to take various actions that promote good health. Thirdly, it reduces the risk of developing diseases, as pessimists are much more likely than optimists to contract various types of diseases. Fourthly, it reduces the negative effects of stress, which is one of the main factors in the development of many diseases, including circulatory system diseases. Optimists are less likely to develop these diseases because they cope much better with stress and have much lower levels of cortisol (the stress hormone) (36–49).

Future studies should include larger groups of women and consider other factors that may additionally contribute to dispositional optimism.

## Conclusion

In general, we have found differences in the dispositional optimism between women from Poland, Belarus, Greece, Turkey, and Russia The studied women, had average and medium levels of dispositional optimism. More optimistic women were pregnant from Belarus and Russia. The dispositional optimism in pregnant women negatively correlated with depressive symptoms and mental health. In the control group, dispositional optimism positively correlated with life satisfaction. In none of the countries, having children by pregnant women and the type of last delivery affected their current level of life optimism.

## Limitations of the study

We did not calculate the sample size.
The study was conducted during the COVID-19 pandemic, which could impact the optimism levels among the studied women.
The participants in this study were not random. This can lead to a biased sample that is not representative of the studied populations.
Due to the differences in the residence structure of female respondents from different countries, depending on the geographical region where the surveys were conducted, it was assumed that in the 21st century, the place of residence does not significantly determine the quality of life and the influence of this factor should not be considered further.
In Russia and Belarus, and to a slightly lesser extent in Greece, the surveys for non- pregnant women were conducted mainly among young people, which may have significantly influenced the results.

### The strength of the study

Our study contributes new knowledge about the dispositional optimism in pregnant and non- pregnant women from five different cultural countries: Poland, Belarus, Greece, Russia, and Turkey. We have compared the dispositional optimism in pregnant, post-partum, and non- pregnant women. We have analyzed the dispositional optimism in women with correlation with life satisfaction, depressive symptoms, and mental health.

### Implications

There is a need for physicians and nurses/midwives to become more involved in the health education of pregnant women regarding a health-promoting lifestyle. This education should consider patients’ resources, especially the level of dispositional optimism, as a factor that strengthens motivation and consistency in following medical recommendations. It is important to strengthen women’s reservoirs of dispositional optimism, even though it is a relatively fixed attribute. After all, it cannot be ruled out that in a difficult situation such as pregnancy, fear of childbirth and childbirth may, on the one hand, decrease and, on the other, intensify under the influence of health educators’ actions. These findings may have important implications for pregnant and postpartum women. Perinatal screening for depression is common, but monitoring the level of optimism is not used in clinical practice. We suggest there is also a need to assess the level of optimism in primary care antenatal services. Moreover, we have found dispositional optimism in the LOT-R scale generally was positively correlated with life satisfaction and negatively correlated with mental health, depressive symptoms, anxiety, and somatic disorders. We suggest that knowing the importance of optimism in pregnant and post-pregnant women can facilitate a more elevated detection of women’s mental health disorders.

## Data availability statement

The raw data supporting the conclusions of this article will be made available by the authors, without undue reservation.

## Ethics statement

The studies involving humans were approved by the Bioethics Committee APK.002.587.2021, Medical University of Białystok, Poland. The studies were conducted in accordance with the local legislation and institutional requirements. The participants provided their written informed consent to participate in this study.

## Author contributions

AK-B: Conceptualization, Data curation, Formal analysis, Investigation, Methodology, Writing – original draft. AS: Data curation, Investigation, Writing – review & editing. NK: Data curation, Investigation, Writing – review & editing. LH: Data curation, Investigation, Writing – review & editing. IA: Data curation, Investigation, Writing – review & editing. DC: Data curation, Investigation, Writing – review & editing. LK: Data curation, Investigation, Writing – review & editing. AT: Data curation, Investigation, Writing – review & editing. KK: Data curation, Investigation, Writing – review & editing. NW: Conceptualization, Methodology, Supervision, Writing – review & editing.
